# Experiences of Upper Limb Somatosensory Retraining in Persons With Stroke: An Interpretative Phenomenological Analysis

**DOI:** 10.3389/fnins.2019.00756

**Published:** 2019-07-24

**Authors:** Megan L. Turville, Johanne Walker, Jannette M. Blennerhassett, Leeanne M. Carey

**Affiliations:** ^1^Occupational Therapy, School of Allied Health, Human Services and Sport, College of Science, Health, and Engineering, La Trobe University, Melbourne, VIC, Australia; ^2^Neurorehabilitation and Recovery, Stroke, Florey Institute of Neuroscience and Mental Health, Melbourne, VIC, Australia; ^3^Occupational Therapy Program, School of Health and Social Development, Faculty of Health, Deakin University, Geelong, VIC, Australia; ^4^Department of Physiotherapy, Austin Health, Melbourne, VIC, Australia

**Keywords:** stroke, somatosensation, therapy, rehabilitation, interpretative phenomenological analysis

## Abstract

**Purpose:**

The aim of this study was to explore experiences of upper limb somatosensory discrimination retraining in persons with stroke.

**Methods:**

A qualitative methodology was used within the context of a randomized control trial of somatosensory retraining: the CoNNECT trial. Participants in the CoNNECT trial completed a treatment program, known as SENSe therapy, to retrain upper limb somatosensory discrimination and recognition skills, and use of these skills in personally valued activities. Eight participants were interviewed on their experience of this therapy. Data were analyzed using Interpretative Phenomenological Analysis (IPA).

**Results:**

Five themes represented participants’ experiences of upper limb somatosensory retraining after stroke: (1) loss of sensation and desire to reclaim normality; (2) harnessing positivity in the therapeutic relationship and specialized therapy; (3) facing cognitive and emotional challenges; (4) distinct awareness of gains and differences in bodily sensations; and (5) improved functioning: control and choice in daily performance. Persons with stroke experienced somatosensory retraining as a valuable treatment that provided them with sensory and functional gains.

**Conclusion:**

Upper limb somatosensory retraining is a treatment that persons with stroke perceived as challenging and rewarding. People who have experienced stroke believed that somatosensory retraining therapy assisted them to improve their sensation, functional arm use, as well as daily performance and participation in life.

## Introduction

Stroke happens unexpectedly with rapid adverse effects on brain function (Sacco et al., 2013). Global estimates reveal 62 million people survive stroke, with numbers continuing to increase (Strong et al., 2007; Mukherjee and Patil, 2011). Stroke causes various impairments leading to disability (Sturm et al., 2002; Hartman-Maeir et al., 2007; Strong et al., 2007; Mendis, 2012). In particular, somatosensory impairment occurs commonly after stroke; approximately 50% of people cannot detect or interpret bodily sensations in the upper limb, such as touch or body position (Carey and Matyas, 2011; Kessner et al., 2016). Somatosensory loss is associated with reduced motor ability and poor functional outcomes after stroke (Blennerhassett et al., 2007; Tyson et al., 2008; Meyer et al., 2014, 2016).

Two recent qualitative studies provide insight into the experience of upper limb somatosensory loss in people who have experienced stroke (Connell et al., 2014; Doyle et al., 2014). In these studies, participants report that somatosensory loss impacts negatively on their performance, roles, and participation in life situations (Connell et al., 2014; Doyle et al., 2014). Some cannot express what it feels likes to have reduced or absent sensation in the upper limb post-stroke and instead describe sensory loss in relation to movement difficulties (Connell et al., 2014). In general, somatosensory impairment is reported to be an unpleasant physical and emotional experience, and people live with uncertainty regarding whether to use their sensory affected arm in daily life (Connell et al., 2014; Doyle et al., 2014). People remain hopeful that their sensation will return after stroke, yet they receive minimal rehabilitation to treat sensory impairment (Connell et al., 2014; Doyle et al., 2014). If rehabilitation occurs, treatment generally involves strategies to compensate for sensory loss (i.e., use of vision or use of the ‘unaffected’ arm). Never-the-less, people who have experienced stroke value treatment that improves somatosensation (i.e., remediates deficits) (Doyle et al., 2014), such as somatosensory discrimination retraining.

Quantitative evidence shows somatosensory discrimination retraining can help people improve their sensation after stroke (Carey, 1993; Carey et al., 1993, 2011a; Yekutiel and Guttman, 1993; Byl et al., 2003, 2008; Carey and Matyas, 2005; Turville et al., 2019). For example, an active remedial approach to sensory discrimination retraining commonly involves learning-based tasks that focus on sensory discrimination and recognition skills (Yekutiel, 2000; Carey et al., 2011a). Principles of retraining originate from theories of perceptual learning and brain recovery; treatment harnesses peoples’ potential for neuroplasticity and learning after stroke (Carey, 1993, 2012b; Yekutiel, 2000). As a consequence, sensory retraining may be a demanding and intense treatment. Previous qualitative studies have focused on the experience of somatosensory impairment, yet no study has specifically investigated experiences of somatosensory discrimination retraining in persons with stroke as a method for upper limb somatosensory recovery.

We therefore aimed to investigate experiences of people with stroke participating in a program of somatosensory discrimination retraining that is based on principles of neural plasticity and learning and designed to help the person with stroke regain a sense of touch. Our focus was on better understanding the facilitators, challenges, and self-perceived changes following upper limb somatosensory retraining post-stroke. Specifically, we wanted to understand: (a) How do persons with stroke perceive and describe their experience of somatosensory discrimination retraining?; (b) What motivates persons with stroke to participate in somatosensory discrimination training?; (c) What variables do persons with stroke perceive as facilitating or limiting their ability to learn and/or perform during somatosensory discrimination retraining?; and (d) What changes do persons with stroke experience following upper limb somatosensory retraining?

## Materials and Methods

### Setting and Study Design

This study recruited persons with stroke with somatosensory impairment who had participated in a randomized control trial of upper limb sensory retraining - the CoNNECT trial (Connecting New Networks for Everyday Contact through Touch) (Carey et al., 2011b). The CoNNECT trial is registered with the Australian New Zealand Clinical Trials Registry (ACTRN12613001136796). The current study was approved as an amendment to the existing ethics approval for the CoNNECT trial, and was obtained from Austin Health and La Trobe University Human Ethics Committees. As part of CoNNECT, participants retrained upper limb sensory discrimination and recognition skills in tactile, proprioception, and object recognition modalities using specially designed training tasks and perceptual learning protocols (Carey, 2012a). Participants also applied sensory retraining principles in relation to two self-selected tasks they considered important in their daily life but had difficulty with due to sensory impairment, e.g., using a wallet or using a fork when eating. This sensory discrimination retraining program is known as SENSe (Carey et al., 2011a).

SENSe therapy is based on seven key principles that are operationalised as follows: (1) selecting specially designed tasks that involve graded somatosensory discrimination of tactile, proprioceptive and haptic object recognition attributes, such as texture surfaces that vary in degree of roughness, friction or pattern, flexion/extension positions of the wrist in space, and object pairs that vary specifically in shape, size, texture, temperature, weight, hardness and function; (2) using goal-directed attention to explore the sensory task with vision occluded and making a perceptual discrimination choice, e.g., about whether the stimulus feels the same or different, or the relative position (angle) of the upper limb joint; (3) receiving feedback from the therapist about the accuracy of sensory discrimination choice (outcome) and method of exploring the sensation (performance); (4) using vision and the experience of feeling the sensory stimulus with the ‘unaffected’ limb in order to calibrate sensation in the affected limb; (5) knowing what to expect to feel (i.e., deliberate use of anticipation) in subsequent sensory discrimination trials; (6) repeating discrimination trials, with task difficulty progressively increased over time; and (7) using a matrix of varied stimuli and training conditions, i.e., learning the above principles within a variety of tactile, proprioception, tactual object recognition and functional tasks, so skills can be transferred to new tasks and situations performed outside of formal training sessions. Further information about this treatment program is detailed in the Carey et al. (2011a) randomized control trial publication and in the SENSe training manual and DVD for therapists (Carey, 2012b). A YouTube video of how the training principles are operationalised is also available online^1^.

### Methodology

A qualitative methodology guided this study; more specifically, Interpretative Phenomenological Analysis (IPA) (Smith et al., 2009) was used to understand, in detail, each participant’s unique view of somatosensory retraining after stroke. Phenomenology is concerned with how people perceive their lived experiences (Merleau-Ponty, 1962) (i.e., phenomenology of perception). As such, IPA seemed particularly relevant for this study to investigate the perceptive process and changes people experience with treatment that addresses an abstract bodily impairment, such as upper limb somatosensory loss. Further, interpretative analysis would enable a depth in understanding the process of somatosensory retraining and potential changes in sensory impairment (Smith et al., 2009). IPA has previously been used in the field of neurology and to understand patient’s treatment experiences (Smith, 2011).

### Participants and Recruitment

We purposively selected a sample of 14 persons with stroke to contact for potential participation in the current study, out of a current sample of 36 from the CoNNECT trial who had all experienced upper limb somatosensory retraining as part of their involvement in the CoNNECT trial. IPA research typically involves detailed investigation of a small sample (Smith et al., 2009). We therefore planned to sample 6–8 persons with stroke. We elected to identify and contact 14 persons with the expectation that approximately half might be available and willing to be involved. In order to maximize the representativeness of the sample and minimize bias we chose to stratify this sample (Patton, 2002) according to their treating therapist and age. These features were selected for stratification because we wanted to (a) sample the experience of treatment delivery across therapists and ensure the interviewer had not previously treated the participant, and (b) sample a range of ages of participants from the CoNNECT sample that comprised a majority of participants under the age of 65 years. In regard to age, we stratified the sample according to (a) those aged over 65 years and (b) those aged less than 65 years. We sent letters to the stratified subset of 14 possible people inviting them to participate in the study. A total of eight participants replied to this invitation and participated between August 2016 and January 2017.

### Procedure

Participants were engaged in semi-structured interviews to obtain qualitative data. This method is often used in IPA research because it enables rich, detailed data on people’s experiences (Smith et al., 2009). Our interview guide was developed using recommendations from Smith et al. (2009) and is presented as Supplementary Material. Interview questions were open-ended to facilitate a detailed understanding of experience. Other types of questions were also used to clarify participants’ experiences, such as: follow-up, probing, or specifying questions (Steinar, 1996; Smith et al., 2009). Interviews were audio-recorded with permission from participants. Interviews and consent took approximately 50 min and occurred either at participants’ homes or at the research clinic. The interviewers were research occupational therapists with no prior relationship with the participant, yet had experience delivering SENSe sensory retraining. Two researchers (MT and JW) completed interviews and reflections were recorded immediately after each interview to begin the interpretative process.

### Data Analysis

The primary author (MT) conducted the primary data analysis, and to ensure trustworthiness in the analysis process, a second researcher (JW) assisted in thematic analysis of four interviews (i.e., 4/8 interviews). Reflectivity statements were employed as a process of reflexivity, and audit trials as a process of recording decision making during the analysis process, to maintain rigor (Krefting, 1991; Roulston, 2010). The iterative analysis process occurred using the following steps, suggested by Smith et al. (2009):

• Audio recording were listened to and transcribed verbatim into written form.
• Participant transcripts were read with initial noting of linguistic, descriptive, and conceptual comments.
• Initial notes were reviewed and conceptualized as themes, which involved deciding on phrases that reflected the psychological essence of that piece of the transcript.
• Themes were categorized into subthemes and then these subthemes were categorized into superordinate themes (i.e., five main themes of this study). Interview extracts that reflected themes were compiled for each participant. Written post-interview reflections from interviewers (MT and JW) were also consulted at this point to ensure completeness of individual participant themes.
• Data was analyzed according to similarities and differences in themes amongst participants. Superordinate and subthemes were identified as group themes if they occurred in interviews of six to eight participants (i.e., were representative of at least 75% of the sample), and as individual themes if they occurred in interviews from less than five participants (Smith et al., 2009).
• Individual themes were categorized in relation to superordinate group themes and the write-up of results maintained a diverse and detailed idiographic focus (Smith et al., 2009).
• Extracts were included in the results section if they represented group themes or individual variability and depth in experience (Smith et al., 2009; Smith, 2011).

## Results

**FIGURE 1:**
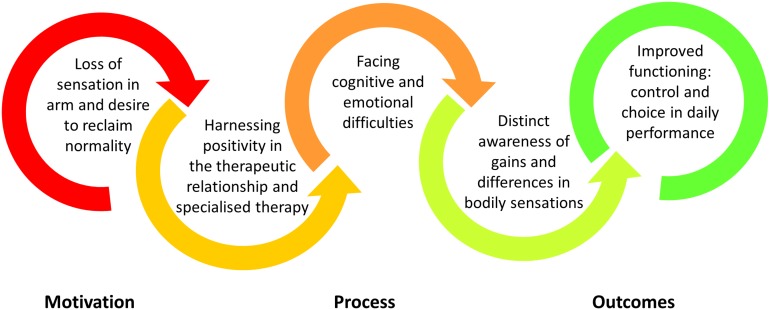
Schematic representation of the main themes that represented participants’ experience of upper limb somatosensory retraining post-stroke.

Findings relate to the experience of eight participants; three females and five males with a mean age of 45 years (*SD* = 11). On average, participants had completed somatosensory discrimination retraining 2.5 years prior to participating in this interview study (*SD* = 1.6 years; Range = 5.5 months to 4.6 years). Additional characteristics of these participants are presented in Table 1. Five superordinate themes represent participants’ experience of upper limb somatosensory discrimination retraining. These themes are schematically represented in Figure 1 and are presented sequentially in the following section.

**TABLE 1:** Participant characteristics.

**Name**	**Affected Arm**	**Time post-stroke (wks)**	**Initial tactile impairment**	**Initial proprioception impairment**	**Initial object recognition impairment**	**Initial motor ability**	**Self-selected tasks involved in retraining**
Michael	Right	82	17.92	23.05	2	38	Using key and stabilizing food
Maria	Left	44	53.54	15.05	41	n/a	Doing up jewelry and hair
Veronica	Left	63	−0.15	34.70	12	62	Typing and brushing hair
Simon	Right	21	8.12	7.60	20	55	Typing and handwriting
James	Right	85	53.03	16.00	19	33	Typing and handwriting
Kevin	Right	16	22.57	11.90	26	45	Using buttons and remote controller
Carlos	Right	29	22.05	18.65	8	31	Using knife and using hammer
Julie	Right	40	15.34	9.95	38	45	Handling money and stirring

*Pseudonyms are used for participant’s names. All participants were right hand dominant. Time post stroke refers to time between stroke and completion of somatosensory retraining program. Initial tactile somatosensory impairment was based on quantitative measurement using the Tactile Discrimination Test (Carey et al., 1997), with an area under the curve score of less than 60.25 indicating impairment in tactile discriminative sensibility. Initial proprioception somatosensory impairment was measured using the Wrist Position Sense Test, with a score above 10.37 degrees indicating impairment in wrist position sense (Carey et al., 1996). Initial object recognition somatosensory impairment was assessed using the functional Tactile Object Recognition Test, with a score below 32 indicating impairment in functional tactile object recognition ability (Carey et al., 2006). Initial motor ability was measured using the upper extremity component of the Fugl Meyer Scale, which has a maximum score of 66 (Fugl-Meyer et al., 1974). n/a means not available. Sensory retraining principles were also practiced in relation to two meaningful tasks chosen by participants – these are indicated in the table for each participant. Consistent with eligibility criteria for the CoNNECT study, participants in this sample did not have: central nervous system dysfunction other than stroke; peripheral neuropathy; nor unilateral spatial neglect.*

### Loss of Sensation in Arm and Desire to Reclaim Normality

Somatosensory impairment evoked an extreme sense of loss for participants, as Carlos states: **“**I don’t feeling nothing, nothing at all […] no sensation at all**”** (Carlos). Participants had lost a vital sensory connection to their body, with some describing complete detachment from their upper limb: “It pretty much was not in my consciousness […] it was pretty much dead on arrival […] there was nothing” (James). The affected arm vanished from people’s known bodily reality. Participants also endured pain (hypersensitivity) in their altered bodily experience. Veronica explains:

I had virtually no touch sensation at all and in the meantime I had and still have got incredible pain through my affected side […] It is very difficult for people to understand the experience that you are having when you can move your hand but you can’t feel, and a big part of the kind of grieving fight is the fight for validation effectively, and when absolutely everything bilateral that you are trying to do is taking enormous effort and causing huge amounts of cognitive fatigue and no-one around you will accept that it is real, you are kind of fighting on both fronts (Veronica).

Upper limb sensory loss may amplify other hidden stroke symptoms, such as fatigue, and impacts negatively on daily task performance, roles, and connection with others. Maria says: “Oh, it was horrible, actually, because I couldn’t really do daily stuff […] I couldn’t hold anything, things were just dropping, yeah so I had nothing, there was nothing there” (Maria). For Veronica, sensory loss affected her central role as a mother – protector of her young*:*

Initially, I couldn’t walk to the playground at the end of my street with my two children holding their hand because I couldn’t feel at all if I was holding their hands because they were little and they were runners. They were too little to have near roads if I wasn’t sure of holding their hands (Veronica).

To provide for his family, Kevin states “I don’t want to be a burden to my family. After the stroke, I need to move on straight away – I’m the head of the family” (Kevin). Overall, participants were confronted with sensory loss and keen to reclaim normality in their body and functioning. Michael reveals the wish to get back to normality as a motivator when asked why he wanted to participate in sensory retraining:

Ah, because, ah, why not give it a go, like, I’ve got nothing to lose […] and if you got no touch, geez […] yeah basically I wanted to get my life back on track and return back to normal (Michael).

Participants had lost so much and, therefore, perceived only gain in participating in upper limb sensory retraining. In addition, participants were grieving after stroke and possibly coped through participating in research and therapy-orientated action, as Julie explains:

I think you just want to get as good as you think you’re going to and that is hard. I mean I was […] too young. I know other people have things when they are very early but you know it changed my life totally. But this I think was one of the best things I had done. I tried a few different things but this I found was really good (Julie).

### Harnessing Positivity in the Therapeutic Relationship and Specialized Therapy

Participants recalled the discrimination tasks and functional retraining they completed as part of this program, with some participants describing the breadth and interest of activities. For example, Simon says:

Um, I think it was multifaceted really, which made it um particularly interesting. We didn’t do exceptionally repetitive tasks necessarily, although there was some repeating of tasks in the sense of assessing differences from yesterday to today for example. It had multiple areas of focus from grid training, to texture training, to object training, um to functional everyday activities like writing, maneuvering objects with my affected limb. So it was really fantastic, it was very holistic approach to functional retraining (Simon).

Discrimination tasks were performed using the learning and neuroscience principles of this program, and participants had declarative and procedural knowledge of treatment principles. Despite this, participants spoke most about the therapeutic milieu in which these principles were enacted. Participants trusted the therapist knew and would teach them sensory retraining principles. For some participants, such as Veronica, education and trust were essential in preparing to participate in sensory retraining.

I couldn’t see the direct cause and effect of how they were going to help me, they seemed a bit abstract and I knew I was going to have to put in a lot effort to make gains and so I was sitting there asking umm polite doubting questions […] Yeah, what does this do and how does it work, and it was like ‘okay now I get it and cool off we go’ (Veronica).

With trust forming, participants used the therapeutic relationship to further understand and cope with learning involved in retraining upper limb sensation. “Look, we all done it. The help of the therapists because by myself, forget it.” (Carlos). Various aspects of the therapeutic relationship were uniquely valued in the learning context of sensory retraining, such as:

• Collaborative effort: “We both worked so hard, so, so, so hard. Like we really both gave it our all” (Veronica).
• Goal-focused: “This is a goal and we will do it. She is very caring […] Yeah, like she is a lovely lady and um yeah and um she’s um helping me every step of the way” (Michael).
• Shared knowledge: “Not only, was doing the activities as important but also from my point of view was understanding, and the therapist was really good at explaining why we were doing certain things […] and that was fantastic” (Simon).
• Problem solving resources for retraining: “Asking her where she got that from and she would say ‘oh yeah I walk around thinking oh yeah that would be good’ and you just don’t think about that” (Julie).
• Encouragement and motivation: “So yeah we did a lot […] really encouraging and would always make me feel welcome and would tell me how I was going […] and I think pretty much she kept me going” (Maria).
• Emotional support: “Positive, positive. There was always an element where I was really asked how I was feeling in the training and that was fantastic […] They were very diligent with that, always conscious to how I felt with different activities” (Simon).

Participants appreciated the one-on-one therapeutic relationship developed during sensory retraining. They felt positive about retraining within a therapeutic relationship that focused on quality practice, scientific information, goal achievement, and personal support.

### Facing Cognitive and Emotional Challenges

Retraining involved demanding and intense work. Participants’ cognitive and coping resources were challenged during treatment; “By the end of it I was like ‘phew’ so exhausted” (James). Participants struggled to sustain their concentration on sensory tasks, yet they believed this challenge was necessary if they were to change how their brain understands bodily sensory information.

It was from a completely different type of thinking and processing than I had ever been used to […] It was always a positive thing, it was challenging but I wouldn’t have changed it, I knew it was working then. […] I used to be exhausted afterward, my brain was just fried […] it was full on […] I think, the way I understood it was that you are just rewiring your brain to connect things again and from your brain to the nerves in your arm so they work properly again (Maria).

Participants were committed to putting in effort to attend to sensory discrimination tasks. Some participants were in awe of their brain and sensation during this process.

When I first started, with the first, as we went on each session I get very tired, very, very tired and then I would not be very good and I would know I was getting enough […] It’s amazing how much the brain says everything. I was just amazed how much […] I don’t think I realized how much the change had occurred and it is ongoing. That something is gone but then something else is on (Julie).

Participants spoke of an understanding of neuroplasticity prior to retraining; however, as Simon outlines the difference between knowing about brain plasticity at an intellectual level versus lived experience. “Until you’re in that position and going through it personally, um, I don’t think you can really appreciate the complexity of it” (Simon). Participants were engaged in a dynamic and complex learning process during sensory retraining, with two participants likening retraining to effortful child development. “Just rewiring everything, like starting again, like a child how they start to learn things that was how I was doing that. […] yeah were as they (children) soak it in, I had to work at it” (Maria). “Because I believe like a little kid to have to do everything again” (Carlos).

Participants coped with the demands of sensory retraining while processing a range of emotions, such as sadness, anxiety, and frustration. Such negative emotional responses seemed to originate from grief following sensory impairment and stroke, and expectations of recovery, learning, and/or performance. For example, while in a perceived regressed stage of development, Carlos describes emotions of sadness during retraining: “Yes, because when the people ask me if I feeling or what I have in my hand, or this, oh and it makes me so upset because I can’t feeling” (Carlos). Carlos initially felt anxious and uncertain about the possibility of sensory recovery with discrimination retraining, and he coped with expressing skeptic thoughts and possible avoidance behavior.

I’m going there [sensory retraining] and I sure nothing going to happen. I just think to myself what am I doing here you know […] It look a little bit stupid what I’m doing there […] The therapists explained things very well just the way I feeling cos I never had a stroke before, thank god for that anyways. I don’t know the way things work you know (Carlos).

Kevin also faced his own challenges with grief and sadness; he coped with acceptance self-talk: “Yes, I am not upsetting myself because I need to accept that I have a problem on myself, I’m a stroke survivor. I need to learn things again from 0 to 100” (Kevin). Other participants reported frustration as a common emotion experienced during retraining. Simon wanted fast results and says:

That was my own frustration in that I wanted to achieve the results quicker than my body and brain would allow […] It didn’t deter me, certainly some frustration around it […] It was a constant challenge for me personally (Simon).

In contrast, Julie narrows her frustration to the perceived right or wrong nature of discrimination tasks:

Oh it was really hard […] I think cos I couldn’t feel. Then we used to have a guess […] You know you hate being, not that you are suffering, but I like to be right […] When we would do the grids I would think ‘argh oh not now’ that’s because I don’t like it getting the better of me (Julie).

Overall, some negative emotions were felt during sensory retraining when experiencing: the effort involved in learning-based brain plasticity and perceptual discrimination tasks, the losses associated with life changes after stroke, and the desire to recover quickly. Some participants also experienced physical and communication challenges during sensory retraining, such as: persistent pain (2/8), increased muscle tone in upper limb affecting object manipulation and proprioception (2/8), and intermittent difficulties with receptive and expressive communication (2/8).

### Distinct Awareness of Gains and Differences in Bodily Sensations

Participants held a positive view of sensory retraining as a treatment to remediate upper limb sensory deficits; all felt their sensation improved with retraining. Sensory gains appeared striking and participants struggled to convey the extent of improvements within the constraints of language. “When I started I had no clue they were different and by the end I was picking everything up […] Um, so, so, so the changes that I experienced in terms of my sensing were pretty remarkable” (James). Michael uses an analogy to explain the change in his sensation:

Yeah, 100%. Like, yeah, it just like when I didn’t have the retraining to when I done it, it’s like chalk and cheese, just, chalk and cheese […] It done wonders for me, like, um, in sensation. […] It’s not 100% but I’ve really got gains of it and I think it will never be 100%. Yeah, like, um from when I started the program to when I finished I’ve made a huge gains (Michael).

Participants described sensory improvements with reference to sensation still not being normal, like before the stroke. Veronica and Simon inform:

No question whatsoever, I have, um, immeasurable greater access to touch sensation and proprioception information than what I had when I started the therapy. The difference that it made for me is indescribable. Um, it is like for a blind person their eyes move but they don’t see, it is that, your hand moves but it doesn’t see. And the difference in what you can do when you can feel something as opposed to not feel something just is indescribably different in life […] I still have imperfect touch (Veronica).

Phenomenally so […] I think what it allowed me to do was relearn how to feel as best as I could on my affected side, especially my arm […] The training has helped that to improve, is it back to pre-level, not completely (Simon).

Participants expressed intimate knowledge about sensory gains and losses after retraining. Participants appraised sensory improvements in relation to their life-long understanding of bodily sensations. At an individual level, Michael indicates that the process of retraining revealed the extent of his sensory loss post-stroke, which further defined the context from which he perceived sensory gains. “Like you don’t know how much you’ve lost until you’ve done the sensation course and it’s like ‘wow, have I lost that much?’ […] Yeah it puts it in your brain how much you can’t do” (Michael).

Sensory improvements motivated ongoing effort in retraining. In the following extract, we hear how improvements dissolved Carlo’s initial ambivalence about retraining:

When I start see the difference I’m so happy going there. I feeling happy, feeling happy every time I going there, I don’t know why […] when I start feeling something it doesn’t matter the time […] The therapist ask me what I have in my hand, you know, and because the feeling is not much and I tell exactly what I have in my hand and, oh, I feeling so good, you know (Carlos).

Sensory improvements were felt not only during the course of therapy but also after formal treatment. “Sensation, no I think I’m still going. It is only very small but it is still improving for me” (Julie). Some participants believed that sensation no longer required as much conscious attention after retraining. “But it sort of gets stuck in your brain, like how it feels, you don’t even like realize that” (Michael). Some participants additionally believed that ongoing sensory gains took time and required dedicated practice of somatosensory retraining principles in daily life.

That was really the key, to understand the principles behind it and transfer that into practice and that certainly helped me to achieve some positivity […] It has become part of my routine, especially things like transferring what I feel on my good side and critically thinking about that and trying to understand what feels it like on my good side and then transferring that to my affected side and trying to feel the same ridges on a piece of clothing, or top, or whatever […] But if I do continue, then there is no reason why it can’t improve albeit slowly over time (Simon).

Simon and Carlos believed that increased arm use was also the catalyst for further sensory gains. “I think it (sensation) is better now. Um, but that is just based on my everyday activities because I even catch myself out because I’m actually incorporating my right hand more now” (Simon). “Yeah because I have never stopped, like I use my hand. That is why I’m feeling more things in my hand” (Carlos).

### Improved Functioning: Control and Choice in Daily Performance

Sensory improvements related to functional improvements; retraining helped participants to use their arm more in daily tasks. Participants improved their performance of meaningful daily activities, such as: eating, grooming, dressing, cooking, exercise, driving, work, and gardening. “I always use my right (affected) hand. In our daily life, sensory retraining does a lot of things for me […] I can do all the things at work, at home, the driving” (Kevin). “It helped me like getting dressed, cos like you have to use both hands to pull up your pants and just day to day stuff like driving, just feeling the steering wheel, like cutting up food” (Michael). This time, Carlos uses an analogy in his attempt to explain the extent of difference he experienced in his function after sensory retraining:

Much easier, everything is different, unbelievable. It’s like a glass of water and a glass of wine, but its true […] always I play with my hand […] and now I go for walk and if I see a coin I pick it up (laughter) (Carlos).

Following sensory retraining, participants perceived their arm was more useful in completing daily activities, with the return of spontaneity and playfulness in arm use. Participants owned renewed control and confidence in their arm and this allowed them to choose (i.e., problem solve) how daily tasks could be performed.

I think there is a bit of spontaneity to my right hand but at the same time I can quickly recall the spontaneity of my brain saying ‘just get that tissue with your left hand’ and so I have to pull it back at that stage and ‘hang on, I can do it with my right hand.’ So it is a little bit of both […] then particularly during participating in the study and this time post I’m more reticent to say ‘no; no I can do it.’ It’s the challenge that I set myself (Simon).

Encouraging self-talk promoted ongoing arm use after retraining. Other strategies for arm use and task performance involved extra time to focus and reassurance that the ‘unaffected’ hand can assist if necessary.

When I do things, especially with my affected right hand, I always remember not to rush. Do it slowly, slowly until you do it […] when I’m focusing using my right hand I can do it well. […] Always if my right hand is not working very well I have another hand to help (Kevin).

Like Kevin, most participants stated they needed extra time to focus during task performance because sensation was still vulnerable to other competing stimuli and demands (e.g., noise, fatigue). As Simon reflects: “Through the training […] there is no other external stimulus distracting you so it is different outside of the therapy room” (Simon). Participants indicated that sometimes it was not possible to arrange extra time for task practice and performance using the affected arm because of external pressures; however, they believed this strategy of extra time in daily routines was worth prioritizing and helpful when implemented. Extra time for task practice and performance assisted ongoing challenges with specific fine motor tasks. Participants also found it helpful to occasionally share the task load between hands or pass the task to another person for the sake of efficiency.

To be honest I don’t actually really recognize that I’m using my hand now […] On my jacket with a zip, I use this hand to hold it down while this pulls up, or vice-versa. But I will say ‘hold on a sec, while I’ […] In the bathroom I brush my teeth with right hand for half the time, then I carry on with my left hand […] When I use a knife I can cut through a lot of things but sometimes I’ll go, like if I’m having a steak or something I will go I’ll do one now can you cut the rest (to wife) […] the glasses are quite big so I use this (unaffected) hand. I’m just scared I think.’ (James).

Some participants limited use of their hand if they perceived damage may occur or if they had an alternative efficient resource available, such as typing (technology) instead of handwriting. Simon informs in relation to work:

Given that nowadays we don’t have to write too much anyways, […] so I’ll type notes on an iPad and I think with technologies today there is no reason why that should be a limiting factor for anyone […] I think that being back at work was therapeutic, absolutely. (Simon).

As Simon indicates, this participant group connected strongly with their working identity; return to work was an important motivator for sensory arm use. Half of the participants specifically reported that sensory retraining assisted them to return to work:

Particularly at work I’ve improved since the beginning of program […] you know when I started I was just doing little basic pieces of work, right now I’m doing bigger jobs. I’m actually a [job title] (James).

It gave me skills for everything I need in my job, my daily work […] I felt so proud and so grateful […] I don’t know what I would be like without it and I don’t want to think about that (Maria).

Sensory retraining positively influenced participant’s performance and participation in daily life. In the final section, emotional healing through doing is explained by Veronica – carer of her young:

The improvement is out of sight, and what that means in everyday life is also out of sight […] The things I needed to do just weren’t that exhausting […] I was able to do many, many, many things that my children were saying “mummy, can you?” “Mummy, I’ve threaded the beads, now can you tie the knot for me?” […] being able to look after myself, my kids, get on with life, make meals, hang out washing stuff, for me giving me back that meditative connection with handcrafts in emotional recovery was an amazing gift. So, the blockages for people that it can unblock are not obvious. (Veronica).

## Discussion

The purpose of the current study was to gain an understanding of how people who have experienced stroke and impaired sensation perceive their experience of upper limb somatosensory retraining, including: the motivators to participate in this therapy; the factors that facilitate or hinder their ability to learn and/or perform during retraining; and the perceived changes that occur as a result of this treatment. Five main themes emerged from participants’ experience of upper limb somatosensory retraining: (1) loss of sensation in arm and desire to reclaim normality; (2) harnessing positivity in the therapeutic relationship and specialized therapy; (3) facing cognitive and emotional challenges; (4) distinct awareness of gains and differences in bodily sensations; and (5) improved functioning: control and choice in daily performance. These themes are discussed with reference to clinical literature and the philosophy of phenomenology (study of the essence of perception) (Merleau-Ponty, 1962).

Persons with stroke perceived and described intense differences in upper limb somatosensation after stroke. We, therefore, found our results contrasted the Connell et al. (2014) IPA study that discovered people had limited awareness and/or difficulties describing stroke-related sensory impairment. Participants in the current study were seeking and had engaged in treatment for sensory impairment and were a younger sample of participants (i.e., less than 65 years of age); hence this sample may reflect a different subset of people with stroke compared to those interviewed in the Connell et al. (2014) study who were aged 65–75 years, with one participant aged 45 years. Some participants reported increased awareness of their sensory impairment during training. Most participants described upper limb impairment as distressing; a common thread across other studies (Barker and Brauer, 2005; Connell et al., 2014; Doyle et al., 2014). The invisible nature of upper limb somatosensory loss appeared to add to the grief and distress for some participants in our study. Severity of upper limb sensory loss varied amongst participants; despite this, all felt distinct somatosensory loss that impacted on their connection to self, others, and the environment.

From a phenomenological perspective, some participants may have initially lacked a sense of ownership of their sensory-affected arm. A sense of ownership relates to “a sense that it is I who am experiencing the movement or thought” (Gallagher, 2005, 173); this fundamental component of self can be affected when afferent connection in the body is impaired (Gallagher, 2012a,b). In addition, participant’s sense of agency was disrupted after stroke-related sensory loss. Sense of agency refers to the “sense of being the initiator or source of a movement, action, or thought” (Gallagher, 2005, 173); this process occurs pre-reflectively (i.e., akin to automatically), or reflectively as attributions of agency when we consciously think about what we are doing (Gallagher and Zahavi, 2007; Gallagher, 2012a). Participants felt and thought their arm was ineffective, untrustworthy, and even a liability. Participant’s embodied self may have felt threatened, hence remediation therapy (i.e., sensory retraining) was viewed as a desirable and worthy treatment pursuit.

The therapeutic relationship developed during sensory retraining enabled participants to progress positively through sensory retraining; engagement aided success. Findings confirm the value of the therapeutic relationship and client-centered practice as a process of partnership (Law et al., 1995; Polatajko et al., 2015). Our view of engagement fits with recent conceptualisations of this process being co-constructed in the client-therapist relationship (Bright et al., 2015, 2017). Within the therapeutic relationship, either person can influence the other depending on their perception of engagement, skills, attitudes, and behaviors (Bright et al., 2017). While this study did not focus on therapists’ perceptions, findings revealed participants perceived the therapist as having knowledge and skills in sensory retraining, alongside support and care for them as a person. Mutual respect and encouragement aid satisfaction in stroke rehabilitation (Mangset et al., 2008; Peoples et al., 2011); in addition, the therapeutic dyad in sensory retraining requires shared control, knowledge, and responsibility (Yekutiel, 2000). In the current study, participants seemed to value a safe and supportive therapeutic space to begin using an arm that feels different and unpleasant. Development of a strong, trusting therapeutic relationship appears to assist persons with stroke to engage in sensory retraining and use their arm. In addition, valued features of the therapeutic relationship in this specialized therapy may have assisted participants to cope with challenges encountered during the somatosensory retraining process. The experience of processing and filtering relevant somatosensory information along with positive affirmation may have assisted participants in this therapeutic process after stroke.

Upper limb somatosensory retraining involves cognitive and emotional challenges. Participants viewed cognitive effort (i.e., sustained concentration) and resultant fatigue as part of the neuroplasticity process to retrain sensation. Similarly, Signal et al. (2016) found that persons with stroke considered fatigue as a natural part of high intensity exercise. While most of us are familiar with exercise, specific education on learning-based neuroplasticity seems to be required after stroke, and this information on brain recovery was perceived as important to participants during retraining. Participants also experienced retraining in relation to their life narrative and coping styles. At times, participants responded to the demands of retraining with thoughts related to self-expectations and loss after stroke. Such thoughts led to negative emotions of anxiety, frustration, or sadness that presented intermittently during treatment sessions. In stroke care, clinicians must attend to a persons’ physical, psychosocial, and relational health (Kitson et al., 2013); hence in retraining, persons with stroke appear to require time and support in the therapeutic relationship to regulate emotions. Therapists are witness to people’s effort and gains made during retraining, and active listening skills from therapists may be essential in this learning-based process. Overall the therapeutic milieu may interact to mediate the effect of emotional or cognitive challenges, and this can enable persons with stroke to progress positively through and meet the challenges of an intensive somatosensory discrimination retraining program.

Results indicated that somatosensory and functional changes occurred with upper limb sensory retraining. Participants described clear improvements in their ability to interpret bodily sensations, and all had a sense of ownership of their arm. In clinical trials, sensory improvements were found using quantitative measurement before and after somatosensory retraining (Yekutiel and Guttman, 1993; Byl et al., 2003, 2008; Carey et al., 2011a). Retraining was not fully curative of sensory deficits, yet facilitated sensory improvements to the degree that people felt more connection with and use in their arm. Participants interviewed reported using their arm more in daily activities, which is essential for ongoing brain and arm recovery (Barker et al., 2007). Participants felt positive changes in their performance and participation of daily activities, such as returning to work. Return to work indicates success in rehabilitation (Alaszewski et al., 2007; Treger et al., 2007), and generally, persons with stroke view good upper limb recovery as return of sensation and movement, functional arm use, and potential for ongoing improvements (Barker and Brauer, 2005). From this perspective, we consider that participants experienced a good recovery with upper limb sensory retraining. Participants felt positive emotions (i.e., happiness, gratitude, and pride) about sensory and functional changes, which also confirms that retraining aided their pursuit of recovery after stroke. All participants recommended retraining to other people with stroke and potential somatosensory loss.

Findings suggest participant’s sense of agency also changed as a result of sensory retraining. Our pre-reflective sense of agency involves a fundamental feeling of embodiment and perceptual awareness that our actions have influence in the world (Gallagher, 2005, 2012a,b). With the return of sensation, participants naturally felt more in their upper limb and spontaneously used their arm more to perform actions. Reflective attributions of agency (Gallagher and Zahavi, 2007; Gallagher, 2012a) was also enhanced as participants controlled and choose how they would use their arm to perform daily activities. Participants’ used various strategies to achieve task practice and performance, such as: self-talk, allowing extra time, or sharing task load with the other hand and/or another person. Self-talk encouraged arm use and/or prompted slower task performance and practice. Other researchers also reveal the significance of self-talk for ongoing upper limb rehabilitation (Sabini et al., 2013). Participants’ arm use strategies were context specific; our actions are continually grounded in time and situation (Gallagher, 2012b). In sum, participants attempted to do more with increased success after upper limb somatosensory retraining and this appears to have contributed to a renewed sense of agency.

Results relate to persons with stroke who participated in a clinical trial of sensory retraining. It is possible this study involved highly motivated people who related well to health professionals and were cooperative (Maclean et al., 2000). Most participants had some degree of motor ability post-stroke, and most experienced upper limb somatosensory impairment in their right dominant hand (*n* = 6/8), which may have influenced the importance they placed on sensory loss as a problem, and thus retraining as a potential solution. All were between 31 and 61 years of age (*M* = 45), thus results may be more applicable to the growing cohort of younger persons with stroke (Wolf et al., 2009). Despite aiming to include older participants (aged 65 years or older) via a stratified sampling method (Patton, 2002), the majority of participants (80%) from the CoNNECT trial were aged 60 years and under at the time this study was conducted. In regard to the data analysis procedure, trustworthiness was promoted with a second researcher (JW) analyzing 4 of 8 interview transcripts. It is recognized, however, that this process did not occur for all interviews completed in this study and is thus a limitation.

The experience of sensory retraining involved an active remedial (learning based) approach to somatosensory discrimination training. The retraining approach, known as SENSe, has been well characterized (Carey et al., 2011a; Carey, 2012a,b) and was delivered in the context of a randomized controlled trial (Carey et al., 2011a). Future research into the client experience of sensory retraining should occur within a typical clinical environment, including investigation of therapists’ perception of the process of therapeutic engagement that persons with stroke value during sensory retraining. Two therapists conducted sensory retraining with participants and there may be particular characteristics of these therapists that influenced findings.

## Conclusion

In conclusion, somatosensory loss exists as a bodily impairment after stroke, which is visibly concealed, often distressing, and intensely personal. People desire restoration of their embodied self and therefore aspire to complete upper limb sensory retraining. Retraining demands cognitive and emotional energy and to maintain a positive outlook, people value a therapeutic relationship that involves a shared vision of effort and change. People change with somatosensory retraining; they know joy and pride in their sensory and functional gains. Sensation still feels less than perfect after retraining, yet people can connect with and use their arm in ways that provide control, confidence, and choice in daily performance and participation.

In regard to the implications for rehabilitation, people experience meaningful somatosensory and functional gains with a perceptual learning, neuroscience based approach to upper limb sensory discrimination retraining after stroke. People may report a desire to improve their sensation and reclaim normality following stroke, and thus should be offered the opportunity in rehabilitation to remediate somatosensory deficits (i.e., somatosensory discrimination retraining). People manage the demands of somatosensory retraining within a therapeutic relationship that contains trust and support. Therapists need to listen to and validate the person’s experience of somatosensory impairment. From this beginning, the therapist and person with stroke can partner in somatosensory retraining to create change and reflect on gains.

## Author’s Note

LC is the original developer of the sensory retraining program investigated in this study. For this reason, MT and JW were involved in data collection and analysis.

## Data Availability

All datasets generated for this study are included in the manuscript and/or the Supplementary Files.

## Ethics Statement

All subjects gave written informed consent in accordance with the Declaration of Helsinki. The protocol was approved by and conducted in accordance with recommendations of the Austin Health and La Trobe University Human Ethics Committees.

## Author Contributions

MT was involved in the study design, ethics application, recruitment, data collection, data analysis, and manuscript drafting and editing. JW completed the data collection, partial data analysis, and manuscript review. JB reviewed manuscript drafts. LC was involved in the study design, data interpretation at the level of a manuscript review, and manuscript revision.

## Conflict of Interest Statement

The authors declare that the research was conducted in the absence of any commercial or financial relationships that could be construed as a potential conflict of interest.

The handling Editor is currently co-organizing a Research Topic with one of the authors LC, and confirms the absence of any other collaboration.

## Footnotes

1 https://www.youtube.com/watch?v=G9V3I30pn68&feature=youtu.be
