# Supplementary material for: Experiences of Upper Limb Somatosensory Retraining in Persons With Stroke: An Interpretative Phenomenological Analysis
Source: Front Neurosci. 2019 Jul 24;13:756. doi: 10.3389/fnins.2019.00756 (PMC6667678; doi:10.3389/fnins.2019.00756)
Supplement: Supplementary file 1 [file Data_Sheet_1.pdf]

Supplementary Material

Experiences of Upper Limb Somatosensory Retraining in Persons with Stroke: An Interpretative Phenomenological Analysis

Megan L. Turville*, Johanne Walker, Jannette M. Blennerhassett, Leeanne M. Carey

*** Correspondence:** Megan Turville**:** M.Turville@latrobe.edu.au

Interview Question Guide:

- Could you please tell me why you decided to participate in somatosensory retraining?
- Can you describe what you did in upper limb somatosensory retraining?
- Were there things that you learnt during sensory retraining?
- Do you think sensory retraining changed your sensation?
- Do you think sensory retraining changed your daily life?
- Could you describe what it is that helped make those changes?
- Could you tell me about things that helped you to do sensory retraining?
- Could you tell me about things that were a challenge in doing sensory retraining?
- When you think about the things you learnt, can you describe if you use those with your sensation and daily activities now?
- What would you say to other people with sensory loss who may be considering this treatment option?
- Is there anything else you would like to say about your experience of somatosensory retraining?

Supplementary Figure 1. Interview Question Guide used in an Interpretative Phenomenological Analysis of Participants' Experiences of Upper Limb Somatosensory Retraining
